# Reply of Dr. C. N. Peirce

**Published:** 1897-08

**Authors:** C. N. Peirce


### Domestic Correspondence.

### REPLY OF DR. C. N. PEIRCE.

To the Editor:

Sir,—The resolution sent to you, and published on page 482 in July issue of International Dental Journal, purporting to be a copy of the resolution adopted by the Odontological Society of Pennsylvania, on May 8, was not written until three days after the final adjournment of the Society, on Tuesday, May 11, 1897. The secretary of the Society was invited into the office of the gentleman claiming to have originated and presented the resolution, and there and then for the first time was that resolution dictated to the secretary. The resolution published in the previous issue of the Journal is the one offered by myself and adopted by the Society.

Your's truly,

C. N. Peirce.

